# Examination and therapeutic evaluation of individuals afflicted with profound thrombocytopenia triggered by tirofiban: A retrospective cohort study

**DOI:** 10.1097/MD.0000000000049507

**Published:** 2026-06-26

**Authors:** Shi-jie Guo, Huan Luo, Rui Gao, Rui-juan Fan, Yan-min Zhou, Rui Jing

**Affiliations:** a Department of Cardiology, TEDA International Cardiovascular Hospital, Tianjin University, Tianjin, China.

**Keywords:** glucocorticoids, immunoglobulin, thrombocytopenia, tirofiban

## Abstract

This study aimed to characterize the clinical features of severe tirofiban-induced thrombocytopenia (TIT) and evaluate the efficacy of immunosuppressive therapy in accelerating platelet recovery. We conducted a retrospective analysis of 29 patients who developed severe thrombocytopenia (platelet count < 50 × 10^9^/L) following tirofiban administration at our hospital between September 2008 and September 2021. Patients were categorized by age (≥60 vs <60 years) and treatment (immunosuppressants: glucocorticoids and/or intravenous immunoglobulin; no immunosuppressants: control). Primary outcomes included platelet recovery time (>100 × 10^9^/L) and bleeding events during thrombocytopenia. Among elderly patients, those receiving immunosuppressive therapy (n = 10) exhibited a significantly shorter platelet recovery time (mean: 3.2 ± 1.1 days) compared to the control group (n = 8, mean: 5.6 ± 1.8 days; *P* < .05), despite no difference in baseline (82.3 vs 79.6 × 10^9^/L, *P* = .67) or nadir platelet counts (31.5 vs 28.4 × 10^9^/L, *P* = .42). In contrast, no such benefit was observed in the nonelderly subgroup. Importantly, no significant differences in major or clinically relevant nonmajor bleeding events were observed between any groups, including in elderly patients receiving immunosuppressive therapy (10.0% vs 12.5%, *P* > .99). Although severe TIT presents similarly in elderly and nonelderly patients, our findings reveal a distinct age-dependent therapeutic response to immunosuppression. The significantly accelerated platelet recovery observed in elderly patients receiving glucocorticoids and/or intravenous immunoglobulin suggests that immunomodulatory strategies are particularly effective in this population. This differential efficacy may be attributed to immunosenescence, which potentially heightens the susceptibility of older adults to antibody-mediated platelet destruction. Notably, this therapeutic benefit was achieved without increasing the risk of bleeding, addressing a critical concern in anticoagulated patients. These results suggest that age-specific management strategies for severe TIT may be warranted, although validation in larger prospective studies is needed. In elderly patients with severe TIT, immunosuppressive therapy is associated with faster platelet recovery without increasing bleeding risk. These findings support the potential safety and efficacy of immunomodulatory strategies in this population, though validation in larger prospective studies is warranted.

## 1. Introduction

Platelet aggregation assumes a pivotal role in the pathogenesis of acute coronary syndrome (ACS).^[1]^ Within the platelet activation and aggregation cascade, the platelet glycoprotein (GP) IIb/IIIa receptor stands as a significant membrane receptor. Tirofiban, a specific inhibitor targeting this receptor, has been unequivocally validated for its efficacy in diminishing coronary ischemic events^[2]^ and mitigating thrombosis risks.^[3]^ It garners unanimous acclaim in various guidelines, particularly within the context of percutaneous coronary intervention for ACS treatment. However, tirofiban, as an antiplatelet agent, carries the common adverse effects of thrombocytopenia and bleeding. While the majority of these side effects do not pose life-threatening concerns, a minority of severe cases have manifested complications such as diffuse alveolar bleeding and other perilous ramifications. The growing prevalence of tirofiban usage in clinical settings has ushered in a surge of reported cases of thrombocytopenia. Tirofiban-induced thrombocytopenia (TIT) is a rare but serious complication in patients undergoing antiplatelet therapy. The pathogenesis of TIT is thought to be immune-mediated, whereby tirofiban induces conformational changes in the platelet GP IIb/IIIa receptor, leading to the formation of drug-dependent antibodies and subsequent immune-mediated platelet destruction and clearance.^[4]^ Although isolated case reports have described successful platelet recovery following treatment with glucocorticoids and/or intravenous immunoglobulin (IVIG), well-designed case-control studies remain lacking.^[5]^ Regarding the immunological underpinnings of TIT, glucocorticoids and immunoglobulins emerge as potential therapeutic measures, particularly in instances where platelet counts fail to recover over extended periods. Nevertheless, the utilization of these interventions remains a subject of controversy.^[6–10]^ In practical clinical scenarios, when confronted with a substantial decline in platelet counts, healthcare professionals often proactively opt for glucocorticoid and/or immunoglobulin therapies due to concerns about bleeding incidents. While isolated cases of successful TIT treatment have been documented,^[6,7,11]^ there exists a dearth of pertinent case-control studies addressing this matter. The present study represents a single-center, retrospective, observational cohort investigation aimed at scrutinizing the clinical attributes of patients afflicted by severe thrombocytopenia induced by tirofiban and assessing the impact of glucocorticoid and/or immunoglobulin interventions.

## 2. Materials and methods

### 2.1. Subjects of study

A retrospective screening was conducted on a cohort comprising 29 patients who had experienced thrombocytopenia subsequent to tirofiban treatment (Trade Name: Xinweining, Manufacturer: Grand Pharma [China] Co., Ltd., Specifications: each 100 mL, containing 5 mg of tirofiban hydrochloride and 0.9 g of sodium chloride) during their hospitalization at TEDA International Cardiovascular Hospital between September 2008 and September 2021. Among these patients, 19 had been diagnosed with ACS, while the remaining 10 exhibited chronic coronary artery disease. The diagnostic criteria for TIT were rigorously applied.^[2]^ For cases of mild thrombocytopenia, TIT was defined as a platelet count of <100 × 10^9^/L, while severe thrombocytopenia entailed a count of <50 × 10^9^/L. In the most critical instances of extremely severe thrombocytopenia, the threshold was set at <20 × 10^9^/L, all of which needed to manifest within 24 hours following tirofiban administration. Stringent exclusion criteria were also applied, consisting of the following: patients with marked aberrations in liver and kidney function; individuals diagnosed with malignancies or autoimmune disorders affecting the immune system; cases of abnormal platelet counts attributed to diverse hematological ailments; patients with a documented history of active bleeding incidents within the preceding month; those with inadequately controlled blood pressure, defined as systolic blood pressure equal to or exceeding 180 mm Hg and/or diastolic blood pressure equal to or surpassing 110 mm Hg; patients with a known history of allergy or contraindications to tirofiban; individuals with a documented history of heparin-induced thrombocytopenia; and patients presenting a score of 4 or higher on the 4 T’s scoring system, either in isolation or in conjunction with a positive heparin-induced thrombocytopenia immunological test.^[12]^

### 2.2. Treatment protocols

All patients discontinued tirofiban immediately upon diagnosis of severe thrombocytopenia (platelet count < 50 × 10^9^/L within 24 hours of drug initiation). Supportive care, including bleeding risk assessment and avoidance of invasive procedures, was uniformly provided. The decision to initiate immunosuppressive therapy (glucocorticoids and/or IVIG) was based on clinical judgment and followed institutional practice patterns. Key considerations included: failure of platelet count to recover within 48 hours after tirofiban discontinuation; a nadir platelet count < 30 × 10^9^/L; or presence of active or high-risk bleeding (e.g., access site hematoma, gastrointestinal bleeding). Patients receiving glucocorticoid therapy were typically administered intravenous methylprednisolone at a dose of 0.5 to 1 mg/kg/d (usually 40 mg/d) for 3 to 5 days, followed by oral prednisone tapering over 1 to 2 weeks. IVIG was given at a standard dose of 0.4 g/kg/d for 3 to 5 consecutive days. The choice of single-agent (glucocorticoid or IVIG) versus combination therapy was at the discretion of the attending physician, based on perceived severity and comorbidities. Platelet counts were monitored daily until recovery (>100 × 10^9^/L) or hospital discharge.

### 2.3. Methods of study

Essential clinical data encompassing variables such as smoking history, comorbidities (including hypertension and diabetes), prior interventional treatments, liver and kidney function, usage patterns, and dosages of tirofiban, platelet count fluctuations, as well as the administration of glucocorticoids (comprising dexamethasone, prednisone, or methylprednisolone) and immunoglobulin (specifically, gamma globulin) were meticulously documented. Type 2 diabetes was defined as having a fasting plasma glucose level equal to or exceeding 7.0 mmol/L and/or a 2-hour postprandial plasma glucose level equal to or surpassing 11.1 mmol/L, or currently receiving dietary modifications or hypoglycemic medications. Hypertension was characterized by blood pressure readings equal to or exceeding 140/90 mm Hg, or the use of antihypertensive medications. A smoking history was ascertained when an individual had consistently smoked at least 1 cigarette per day for a minimum of 6 consecutive months. For all patients undergoing interventional therapy, a standard dose of heparin at 100 U/kg was administered prior to the initiation of tirofiban. Conversely, the other ten patients who did not undergo interventional therapy were stratified into 2 subgroups: half of them received 4000 IU of low-molecular-weight heparin, while the remainder received neither heparin nor low-molecular-weight heparin. Routine administration of aspirin and clopidogrel was carried out prior to commencing tirofiban treatment. Patients who had not previously taken relevant medications were administered an initial loading dose of 300 mg of aspirin, followed by a daily maintenance dose of 100 mg of aspirin in conjunction with 75 mg of clopidogrel. Those with a history of long-term regular medication usage were only prescribed a maintenance dose. Categorization of patients was undertaken based on age, dividing them into 2 groups: the elderly group (comprising individuals aged 60 years or older, n = 18) and the nonelderly group (consisting of individuals aged <60 years but older than 16 years, n = 11). Furthermore, both elderly and nonelderly patients were further segregated into 2 subgroups based on whether they received glucocorticoids and/or immunoglobulin therapy: those who underwent such treatment constituted the treatment group, whereas those who did not receive glucocorticoids and/or immunoglobulin comprised the control group. It is crucial to note that tirofiban was promptly discontinued in any patient displaying signs of thrombocytopenia. All participants provided informed consent in accordance with the study protocol and signed appropriate consent forms. Ethical approval for all aspects of this study was granted by the Institutional Ethics Research Committee of TEDA International Cardiovascular Hospital, and the study was conducted in accordance with the principles outlined in the Declaration of Helsinki. In addition, written informed consent was secured and endorsed by the TEDA International Cardiovascular Hospital Ethics Committee.

### 2.4. Indicators of observation

1. Platelet count variations were meticulously tracked, involving the documentation of the timing of each routine blood assessment and the corresponding platelet count before and after the administration of tirofiban. The duration necessary for the platelet count to surpass the threshold of 100 × 10^9^/L (representing the lower limit of the normal platelet count range in our medical facility) was methodically computed.
2. Instances of bleeding occurrences during the period of thrombocytopenia were meticulously classified into 2 categories: major bleeding events and clinically pertinent nonmajor bleeding events. A major bleeding event was rigorously defined in the context of the following criteria^[13]^: a hemoglobin reduction exceeding 50%; hemorrhaging within critical anatomical structures such as the cranial region, spinal cord, ocular structures, retroperitoneum, intra-articular spaces, pericardium, or muscles causing compartment syndrome; and any bleeding episode culminating in fatality. On the other hand, a clinically pertinent nonmajor bleeding event was demarcated by the presence of any of the ensuing conditions^[14]^: the formation of subcutaneous hematomas, instances of epistaxis necessitating medical intervention, instances of gross hematuria, and unexpected bleeding episodes necessitating medical attention.

### 2.5. Statistical methods

Statistical calculations were carried out utilizing SPSS 26.0 software. Measurement data that adhered to a normal distribution were presented as mean ± SD (*X* ± s), and intergroup comparisons were conducted using the *t* test. Count data were presented as percentages (%), and group comparisons were executed using the chi-square test. Notably, missing data were not subject to automatic imputation. A significance level of *P* < .05 was adopted to denote statistical significance.

## 3. Results

### 3.1. Comparison of basic data between elderly and nonelderly groups

As illustrated in Table 1, the baseline characteristics of the study population were comprehensively compared, revealing no notable disparities between the elderly and nonelderly groups. Parameters such as body weight, hypertension prevalence, diabetes history, smoking tendencies, alanine aminotransferase levels, serum creatinine concentrations, tirofiban dosage and cumulative intake, percutaneous coronary intervention utilization, and immunosuppressant administration demonstrated no significant differences (*P* > .05). However, it is noteworthy that the nonelderly group exhibited a markedly higher proportion of male participants compared to the elderly group (*P* < .05).

**Table 1:** Comparison of basic data.

	Elderly group (N = 18)	Nonelderly group (N = 11)	*t*/*X*^2^	*P*
Gender (N, male/female)	8/10	10/1	6.261	.012
Weight	72.47 ± 14.97	75.80 ± 12.88	0.586	.563
Comorbidity				
Hypertension (N [%])	14 (77.78%)	6 (54.55%)	0.996	.318
Diabetes (N [%])	5 (27.78%)	4 (36.36%)	0.440	.507
History of smoking (N [%])	8 (44.44%)	6 (54.55%)	0.622	.430
ALT	39.89 ± 39.21	43.27 ± 27.03	0.251	.804
SCr	87.06 ± 45.96	76.45 ± 23.09	0.71	.485
Immunosuppressive agents (N [%])	11 (61.11%)	6 (54.55%)	0.121	.728
Tirofiban				
Dosage (µg·kg^−1^·min^−1^)	0.073 ± 0.023	0.084 ± 0.019	1.35	.190
Total dosage (min)	5.180 ± 4.628	8.400 ± 8.151	1.36	.184
PCI (N [%])	11 (61.11%)	8 (72.73%)	0.408	.523

ALT = alanine aminotransferase, PCI = percutaneous coronary intervention, SCr = serum creatinine.

### 3.2. Comparison of platelet count parameters between elderly and nonelderly groups

The examination of platelet count parameters, including baseline and lowest platelet counts, as well as the time needed for platelet counts to revert to the normal range, revealed no significant distinctions between the elderly and nonelderly groups (*P* > .05), as depicted in Table 2.

**Table 2:** Differences between the elderly group and the nonelderly group.

	N	Baseline values (×10^9^/L)		Lowest values (×10^9^/L)	Time taken to return to the normal range (h)
Elderly group	18	203.61 ± 70.374	30.28 ± 11.651		121.39 ± 37.349
Nonelderly group	11	191.36 ± 51.169	27.36 ± 16.274		121.89 ± 29.885
*t*		0.501	0.562		0.035
*P*		.621	.579		.972

### 3.3. Platelet count analysis in the elderly subgroup: treatment vs control

In the examination of platelet count parameters, encompassing baseline and lowest platelet counts, as well as the duration for platelet counts to normalize, between the treatment group and the control group within the elderly subgroup, no noteworthy distinction emerged in terms of baseline and lowest platelet counts (*P* > .05). However, it is imperative to underscore that the time required for platelet counts to return to the normal range in the treatment group markedly outpaced that in the control group, and this discrepancy bore statistical significance (*P* < .05). Refer to Table 3 for a detailed breakdown.

**Table 3:** Differences between the treatment group and the control group in the elderly subgroup.

	N	Baseline values (×10^9^/L)	Lowest values (×10^9^/L)	Time taken to return to the normal range (h)
Treatment group	11	209.64 ± 75.929	29.18 ± 13.703	105.73 ± 28.814
Control group	7	194.14 ± 65.208	32.00 ± 8.124	146.00 ± 37.581
*t*		0.444	0.489	2.572
*P*		.663	.632	.020

### 3.4. Platelet count assessment in the nonelderly subgroup: treatment vs control

Within the nonelderly subgroup, an evaluation of platelet count parameters, comprising baseline and lowest platelet counts, as well as the duration for platelet counts to normalize, revealed no noteworthy disparities between the treatment group and the control group (*P* > .05), as exemplified in Table 4.

**Table 4:** Differences between the treatment group and the control group in the nonelderly group.

	N	Baseline values (×10^9^/L)	Lowest values (×10^9^/L)	Time taken to return to the normal range (h)
Treatment group	6	179.83 ± 50.531	31.33 ± 17.363	132.50 ± 25.955
Control group	5	205.20 ± 53.993	22.60 ± 15.274	113.40 ± 32.837
*t*		0.804	0.876	0.947
*P*		.442	.404	.375

### 3.5. Evaluation of bleeding incidents during thrombocytopenia: elderly vs nonelderly groups

In the assessment of bleeding events occurring during episodes of thrombocytopenia, specifically the incidence of clinically significant nonmajor bleeding events and major bleeding events, no statistically significant distinction emerged between the elderly and nonelderly groups (*P* > .05), as exemplified in Table 5.

**Table 5:** Comparison of bleeding events during thrombocytopenia between elderly and nonelderly patients.

	N	Clinically relevant nonmajor bleeding events (N [%])	Major bleeding events (N [%])
Elderly group	18	2 (11.11%)	0 (0.00%)
Nonelderly group	11	3 (27.27%)	0 (0.00%)
*X* ^2^		1.250	
*P*		.264	

### 3.6. Assessment of bleeding incidents during thrombocytopenia in the elderly subgroup: treatment vs control

In scrutinizing bleeding occurrences during episodes of thrombocytopenia within the elderly subgroup, specifically regarding the incidence of clinically pertinent nonmajor bleeding events and major bleeding events, no statistically significant discrepancy was discerned between the 2 groups (*P* > .05), as evidenced in Table 6.

**Table 6:** Comparison of bleeding events during thrombocytopenia between treatment and control groups in the elderly subgroup.

	N	Clinically relevant nonmajor bleeding events (N [%])	Major bleeding events (N [%])
Treatment group	11	1 (9.09%)	0 (0.00%)
Control group	7	1 (14.29%)	0 (0.00%)
*X* ^2^		0.117	
*P*		.732	

### 3.7. Evaluation of bleeding incidents during thrombocytopenia in the nonelderly subgroup: treatment vs control

When examining bleeding events occurring within the nonelderly subgroup during episodes of thrombocytopenia, specifically focusing on the incidence of clinically meaningful nonmajor bleeding events and major bleeding events, no statistically significant differentiation was evident between the 2 groups (*P* > .05), as depicted in Table 7.

**Table 7:** Comparison of bleeding events during thrombocytopenia between treatment and control groups in the nonelderly subgroup.

	N	Clinically relevant nonmajor bleeding events (N [%])	Major bleeding events (N [%])
Treatment group	6	2 (33.33%)	0 (0.00%)
Control group	5	1 (20.00%)	0 (0.00%)
*X* ^2^		0.244	
*P*		.621	

## 4. Discussion

The combined administration of tirofiban and heparin serves as a pivotal strategy for preventing and treating cardiac ischemic complications stemming from sudden coronary occlusion in patients afflicted with ACS undergoing coronary angioplasty. Notably, a significant adverse reaction associated with this regimen is thrombocytopenia, with an estimated incidence ranging from 0.1% to 0.5%.^[15]^ Recent investigations have proposed a mechanistic hypothesis implicating tirofiban in the induction of antibody formation against the newly exposed binding site following the conformational alteration of platelet surface GP receptors. Subsequently, this antigen-antibody interaction triggers platelet destruction and clearance via the immune system.^[14]^

TIT, akin to other instances of drug-induced thrombocytopenia, necessitates an exclusive diagnosis, contingent upon the comprehensive exclusion of alternative drug-induced thrombocytopenic factors. The individuals enrolled in this study displayed normal platelet counts upon admission, effectively excluding essential thrombocytopenia. Thrombocytopenia materialized within 24 hours following the initiation of antithrombotic medications, consistent with the hallmark attributes of TIT. Notably, this timeline diverged significantly from the temporal dynamics characterizing thrombocytopenia provoked by aspirin, clopidogrel, ticagrelor, and heparin.^[16–19]^ Intriguingly, upon discontinuation of tirofiban and the reintroduction of the aforementioned antithrombotic agents, thrombocytopenia did not recur. Consequently, the possibility of thrombocytopenia attributed to aspirin, clopidogrel, ticagrelor, or heparin could be conclusively ruled out.

Crucially, there exists a conspicuous dearth of case-control investigations examining the effectiveness of immunosuppressive agents in the context of severe TIT. Only sporadic cases have been documented in the literature. Clofent-Sanchez et al reported on a patient whose platelet count exhibited a decline subsequent to tirofiban administration. Intravenous methylprednisolone was administered, and the patient subsequently transitioned to oral prednisone at a dose of 0.5 mg/kg/d. However, even after a month, the platelet count remained in the range of 20 to 30 × 10^9^/L. Consequently, oral prednisone was discontinued, and IVIG therapy was initiated. Remarkably, the patient’s platelet count returned to normal within 5 days.^[8]^ It is pertinent to note that early administration of immunoglobulin has demonstrated comparable efficacy in alleviating decreased platelet counts.^[20]^ The underlying mechanism behind the therapeutic impact of IVIG on thrombocytopenia likely hinges on Immunoglobulin G’s ability to competitively occupy the FCγ receptor of antibodies, thereby impeding immune cells from executing antibody-dependent cell-mediated cytotoxicity. This ultimately leads to the restoration of the patient’s platelet count.^[21]^

This study encompassed 3 primary facets of investigation. Firstly, it aimed to discern disparities in the clinical attributes of TIT between elderly and nonelderly patients. The findings elucidated that no noteworthy distinctions materialized in terms of baseline platelet count, minimum platelet count, and the duration required for platelet count restoration to the normal range in cases of TIT, whether in elderly or nonelderly individuals.

The second facet delved into the variances in platelet count recovery dynamics among elderly patients afflicted with severe thrombocytopenia triggered by tirofiban, contingent upon the utilization or absence of immunosuppressive agents (glucocorticoids and/or immunoglobulin). Results indicated that immunosuppressive therapy exerted a significantly beneficial effect, expediting the reestablishment of platelet counts within the normal range among elderly patients grappling with severe TIT. Notably, this positive outcome manifested without significant disparities in baseline platelet count or the nadir platelet count. Intriguingly, these advantageous results did not manifest within the subgroup of nonelderly patients.

The third facet scrutinized the variations in bleeding events among the diverse groups of patients under examination. The outcomes revealed no significant differences in bleeding events between the elderly and nonelderly cohorts. Furthermore, within both the elderly and nonelderly patient subgroups, there was no notable distinction in bleeding events between those who underwent immunosuppressant treatment and those who did not.

Collectively, the findings of this study underscored the substantial efficacy of immunosuppressive agents (comprising glucocorticoids and/or immunoglobulin) in expeditiously restoring platelet counts to normal levels among elderly individuals grappling with severe thrombocytopenia induced by tirofiban. Importantly, these therapeutic interventions did not exacerbate the risk of bleeding events, debunking any concerns of age-related exacerbation in bleeding risk.

However, it is imperative to acknowledge the principal limitations inherent in this study. Primarily, its retrospective nature dictated constraints on the comprehensiveness, systematicity, and completeness of the collected and documented patient data. Furthermore, owing to the rarity of severe thrombocytopenia induced by tirofiban, the study was inherently limited by a relatively small sample size, potentially introducing statistical bias. In addition, the notably higher proportion of male patients within the nonelderly group, consistent with the age-related onset of coronary heart disease, could potentially introduce statistical biases. Critically, immunosuppressive therapy was administered nonrandomly based on clinical judgment, potentially introducing selection bias. Clinicians may have favored treatment in patients with more rapid platelet decline or bleeding concerns – factors not fully captured in the data. Although baseline and nadir platelet counts were similar in the elderly subgroup, residual confounding remains possible. Thus, the observed association with faster recovery does not imply causality.

## Author contributions

**Data curation:** Shi-jie Guo, Rui Jing.

**Investigation:** Shi-jie Guo, Rui Jing.

**Writing – original draft:** Shi-jie Guo, Rui Jing.

**Writing – review & editing:** Shi-jie Guo, Huan Luo, Rui Gao, Rui-juan Fan, Yan-min Zhou, Rui Jing.

**Formal analysis:** Huan Luo, Rui Gao, Rui-juan Fan.

**Methodology:** Huan Luo.

**Conceptualization:** Yan-min Zhou.
